# Seeing Like a State, Enacting Like an Algorithm: (Re)assembling Contact Tracing and Risk Assessment during the COVID-19 Pandemic

**DOI:** 10.1177/01622439211021916

**Published:** 2021-06-04

**Authors:** Chuncheng Liu

**Affiliations:** 1 University of California San Diego, La Jolla, CA, USA

**Keywords:** algorithms, surveillance, sociotechnical assemblage, COVID-19, China

## Abstract

As states increasingly use algorithms to improve the legibility of society, particularly during the COVID-19 pandemic, it is common for concerns about the expanding power of the algorithm or the state to be raised in a deterministic manner. However, how are the algorithms for states’ legibility projects enacted, contested, and reconfigured? Drawing on interviews and media data, this study fills this gap by examining Health Code (*jiankangma*), the Chinese contact tracing and risk assessment algorithmic system that serves as the COVID-19 health passport. I first explore the intensive and invisible work and infrastructures that enact and stabilize Health Code’s sociotechnical assemblage. I then show how this assemblage is frequently challenged and destabilized by errors, breakdowns, and exclusions. Facing unintended engagements from heterogeneous social actors, local interests, and power hierarchies, Health Code reassembles into multiple and contradictory assemblages at different periods and social localities. Finally, I examine how people game and bypass the algorithm’s surveillance with their agencies. Recognizing this messiness and heterogeneity contributes to a more nuanced and realistic understanding of states’ use of algorithms, including the risks. Doing so also urges us to rethink the politics of citizenship and inequality in the digital age beyond inclusion.

## Introduction

COVID-19 is the most significant global pandemic in decades and offers opportunities for states to experiment with new digital tools. Many societies have applied various algorithms alongside other information and communications technologies (ICTs) to trace contacts between infectious parties, evaluate risk, and make governance decisions. These choices have advanced the scale and speed of disease surveillance and risk assessment (Ferretti et al. 2020; Park, Choi, and Ko 2020; Shaw, Kim, and Hua 2020; Ting et al. 2020). While most people saw the benefits of these instruments, many have also been concerned about the social implications of these algorithms as potentially justifying invasions of privacy (Park, Choi, and Ko 2020), as a way to transfer accountability and responsibility from the state to individuals (Rowe 2020), and as an expansion of the state’s power over society (Sweeney 2020). These concerns are largely associated with the growing discussions on statecraft in the digital age (Fourcade and Gordon 2020; Rona-Tas 2020) and worries around how new forms of “seeing like a state” (Scott 1999) increase the state’s power over citizens. Facing controversies, physicians, computer scientists, legal scholars, and policymakers quickly turned to ethical and technical discussions of algorithm design, such as employing the minimalist data collection principle, improving the consent process, involving multiple stakeholders, enhancing data protection, and making institutions accountable for the design of their algorithms (Bengio et al. 2020; Ferretti et al. 2020).

While those discussions contribute to our understanding of current controversies around contact tracing and risk assessment algorithms, they often lack detailed empirical investigation. Instead, they assume that the adoption of algorithms in contact tracing, like other state legibility projects, simply increases the state’s ability to see and intervene more in a centralized way. The quick focus on design also made the public debate primarily “an argument over technical architectures” (Sweeney 2020). Yet, as a growing amount of scholarship has pointed out, technical elements only contribute to part of an algorithm’s power and problems (Christin 2020; Seaver 2017), leaving empirical and theoretical questions about algorithms and state legibility unanswered. If the state does see more clearly and more broadly through its algorithmic eye, how does that happen? If not, what fails?

Following science and technology studies’ (STS) tradition of examining how the power of knowledge is established through its utilization by and interaction with broader sociomaterial actors, devices, and institutions, this study moves beyond technical design to conceptualize and analyze algorithms as sociotechnical assemblages. Merging the theoretical developments in social studies of both algorithms and the state, this study provides timely and novel data on how the state’s algorithmic vision is actualized, experienced, adopted, or refuted in action, exploring this vision’s limits while admitting the reinforced legibility it offers. This study uses a Chinese contact tracing and risk assessment algorithmic solution, the “Health Code (*jiankangma*)” mobile application (APP), to explore these dynamic processes based on in-depth interviews, technical documents, and media articles.

Health Code was codeveloped by state institutions and private companies. It serves as the state’s algorithmic eye to identify, document, and evaluate risk during the pandemic. I first show how Health Code is designed and how its sociotechnical assemblage is assembled in society. This process requires the imbrication of multiple layers of infrastructures as well as an intensive, yet largely ignored, mobilization of both human and nonhuman actors. However, this assemblage is constantly challenged and broken by other social actors. I then examine how these breakdowns occur and how they destabilize Health Code. Furthermore, I explore how Health Code varies and deviates from its original design and intention, reassembling with various actors and multiplying into contradictory sociotechnical assemblages. I lastly show various strategies that people adopt to bypass and game the Health Code regime while navigating through these processes. Exploring these messy and interactive dynamics, I reveal how Health Code, together with the general digitalization of everyday life, grants or obscures the state’s vision, advancing the imperative of digital citizenship in a world where the digital gap is increasingly ignored.

## State Legibility through Algorithms

The state is not only a material entity; it is also one of knowledge (Jasanoff 2004; Mitchell 2002; Morgan and Orloff 2017) that requires information about its subjects for legibility and conducts interventions (Dijck 2014; Scott 1999). Technology is critical for this legibility. Novel ICTs have contributed to the state’s ability to conquer new areas of the social world. Scott (2009, xii) argues that distance-demolishing technologies, such as railroads and ICTs, “changed the strategic balance of power between self-governing peoples and nation-states, [thus] diminish[ing] the friction of terrain” and significantly undermining the ungovernable. From this perspective, the modern state is often depicted as a rational, centralized, and stable entity that sees, calculates, and determines the life chances of the people subjected to it (Loveman 2005; Scott 1999, 2009). This conceptualization of the state shows a great affinity with the mainstream perception of algorithms, which, according to Christin (2020), are seen as black boxes in the cloud—opaque yet powerful and hidden behind walls—that rule at a distance.

Algorithms seem to fit the state’s goal and approach of increasing legibility, not only due to their ability to scale up but also due to their tendency to centralize data collection and decision-making (Rona-Tas 2020; Zuboff 2019). This confluent perception of states and algorithms is particularly common in the authoritarian context, where, after a brief period when the Internet seemed to shake the sociopolitical orders, states have quickly and adaptively equipped themselves with ICTs and algorithms that further advance their vision and control of society (Liu 2019; Michaelsen and Glasius 2018). In the democratic context, scholars have also noticed an escalation of algorithmic datafication and evaluation practices across various social contexts, such as India’s biometric identification system, Aadhaar (Singh and Jackson 2017), and predictive policing in the United States (Brayne 2020). Resonating with scholars’ critique of both predigital state visions and algorithms, the state’s algorithmic eyes have been found to be privacy-invading, biased, lacking accountability, and increasingly “black-boxed” (Brayne 2020; Eubanks 2018; Pasquale 2015).

Although algorithms seem simply to increase the state’s ability to see, recent studies of both the state and algorithms urge more nuanced perspectives. Scholars argue that state use of algorithms heralds a transformation in statecraft (Fourcade and Gordon 2020; Johns 2019) that should not simply be analyzed according to the theories developed in the predigital era. Following STS’s tradition of multiplicity and fluidity, scholars studying the state have also called for a move away from totalizing state-centered views to more dynamic and interactive perspectives that are better able to capture how the state sees and acts on the ground (Mitchell 2002; Morgan and Orloff 2017). In this view, the state is not an abstract and all-powerful actor but an assemblage of fragmented institutions, organizations, and social actors that practice and perform (Jasanoff 2004). As for legibility, it is no longer considered to be the state’s taken-for-granted and undivided power. Instead, it is a capacity that requires constant effort to achieve, maintain, and improve. Recent scholars show that the state does not necessarily see more or more clearly because its vision can be blurred, contested, and negotiated through dynamic social processes (Rodríguez-Muñiz 2017; Greenberg 2021). Furthermore, increasingly, state legibility requirements are connected with citizenship and, in many situations, made a prerequisite for people to receive services from the state. As a result, being seen by the state is a status that people, particularly marginalized social groups, aim to achieve rather than to avoid, in contrast to traditional state-centered analysis (Fourcade 2021; Rodríguez-Muñiz 2017; Rona-Tas 2020). This further promotes the value of a theoretical perspective that includes heterogeneous social actors in the construction and operation of state legibility projects beyond “the state,” as well as the dynamic interactions among these social actors beyond the “see/control–resistance” framework.

Similar developments can be found in social studies of algorithms. As a growing number of scholars emphasize, blackbox-focused studies highlight the technical and design parts of algorithms yet leave them decontextualized (Christin 2020; Geiger 2017; Seaver 2017). These scholars have shown that algorithms do not function simply based on codes that articulate the steps for dealing with the input and producing the output. Instead, their functionalities and power lie in how they enact with other sociomaterial actors within broader sociocultural structures to form sociotechnical assemblages (Lee et al. 2019; Seaver 2017). This engagement can happen at two ends. The first end is where the algorithm datafies “raw” reality into structured information that feeds into calculation (Dijck 2014). The second end is where the algorithm’s output is deployed and used in society. At both ends, human and nonhuman actors are enrolled in the algorithmic assemblage (Christin 2020; Brayne 2020) and different infrastructures are imbricated unevenly with each other (Lampland and Star 2009; Singh and Jackson 2017), requiring intensive yet often invisible infrastructures to function (Star 1990; Vertesi 2014). These enrollments and assemblings are not uninterrupted projections of an algorithmic design but, instead, a process that always involves disassembling and reassembling. Planned infrastructures and devices that support the algorithm may break down (Houston, Gabrys, and Pritchard 2019; Vertesi 2014); actors perceive and behave around the algorithm differently (Amelang and Bauer 2019; Liu and Graham 2021); resistance and struggles occur (Chen and Sun 2020); and, in the process, new sociotechnical actors become involved (Lee et al. 2019). These messy, diverse, and interconnected engagements co-constitute the algorithm’s sociotechnical assemblage. As a result, the very same algorithmic design might be performed in multiple distinctive sociotechnical assemblages at different places with diversely different actors, which “can reveal existing priorities within groups, organizations, and fields, as well as their changes over time” (Christin 2020, 10).

Bringing the new developments of both camps of literature together, this study argues against the sense of determinism that prevails in approaches to both algorithms and states. Currently, studies of state legibility mostly focus on how nonstate actors contribute to the construction of the state’s vision in traditional state projects, such as the census, but they are not attentive to algorithms. Meanwhile, studies of algorithms are generally more aware of the agency of private companies than that of the state, partly because most studies are conducted in Western societies with less salient state intervention. This study, therefore, further advances both fields empirically with timely and innovative data. Particularly, this study addresses the use of algorithms in non-Western and nondemocratic contexts, which is currently lacking in the literature. China has been one of the frontrunners of domestic algorithmic surveillance, as well as an exporter of global surveillance technologies (Hou 2017; Liu 2019). However, while it is often described simply as a technodystopia and an all-knowing state in the popular media, there is little close, empirical work on the topic in a scholarly context. By studying how Health Code has been enacted in China during the COVID-19 pandemic, I offer a process-based and more realistic description of how the state sees through an algorithm.

## Method

To conduct this study, I first collected national standard documents and media coverage of Health Code to show how it is designed and intended to work. Then, from April 30 to June 30, 2020, two research assistants and I conducted thirty-eight interviews with Chinese residents who lived in mainland China and had used Health Code in the past two months. Their lived experiences working or interacting with Health Code showed how Health Code is assembled and reassembled in society. In the first wave of the study, participants were recruited through my research assistants’ and my own social ties (*n* = 11). The second wave participants (*n* = 7) were recruited with a snowballing method based on the first wave of participants. The third wave participants (*n* = 20) were recruited from social media posts of a public health NGO and a social media influencer. I specifically diversified the sample based on gender, location, and age for more systematic analysis and relational aspects of the algorithms.^1^

Demographically, my sample contains twenty-three females and fifteen males from fifteen different provinces in China, ranging in age from eighteen to fifty-five (mean: twenty-nine). Details of the interviewees are in the Appendix. Except for one participant, all interviewees’ Health Code risk status had been low risk (green) before our interview. Twenty-one of them traveled among different cities during the pandemic and used more than one Health Code system. Interviews were conducted online through WeChat (a Chinese message APP) in Mandarin Chinese. The interviews lasted thirty to ninety minutes. All interviews were recorded with consent and then transcribed to text.

All the data were organized and analyzed in MAXQDA inductively. First, I read through all of the material with unstructured coding. Then, I reviewed, revised, and reclassified the unstructured codes into different themes and stages. I organized the findings in a process-oriented order to construct a social life of the Health Code. Following Jasanoff’s (2004) distinction between emergence, stabilization, controversies, and adjustment, I show how Health Code’s sociotechnical assemblage is assembled and stabilized, after which is meets challenges, breaks down, and generates controversies, which are followed by stages of adjustment and reassembling. These stages do not happen in a perfect chronological order; they can happen in different temporalities and overlap with each other.

## Assembling Health Code

### Health Code in Design

Health Code is not a singular system that collects data and determines risk status for all Chinese citizens. Although the technical solutions are almost the same, most cities have independent systems. Starting with the first Health Code released by the Shenzhen government and Tencent on February 9, 2020, almost all Chinese cities acquired a Health Code system by the end of March, running on WeChat, Alipay (a mobile payment APP), or as an independent APP. Gradually, the central government and the provincial governments released their Health Code systems. In many places, national, provincial, and municipal systems do not replace each other but coexist and are used in different scenarios. Since April, China’s COVID-19 outbreak has been mostly under control, yet the use of the Health Code is still being enforced in many cities.

According to the national standard documents, a Health Code system should collect four kinds of data: (1) personal data (name, gender, ID card number, phone number, etc.); (2) personal health data of the day (body temperature, symptoms, high-risk people contact history, etc.); (3) visit histories; and (4) health status data such as testing results. One needs to initiate Health Code with his or her real-name information and complete an epidemiological questionnaire that covers the past two weeks’ travel history, COVID-19-related symptoms, and contact history with those who have been COVID-19 infected. In some cities like Shanghai, facial recognition data are also needed. Based on different municipal governments’ requirements, people may have to update some questionnaire responses daily or never again.

**Figure 1.**
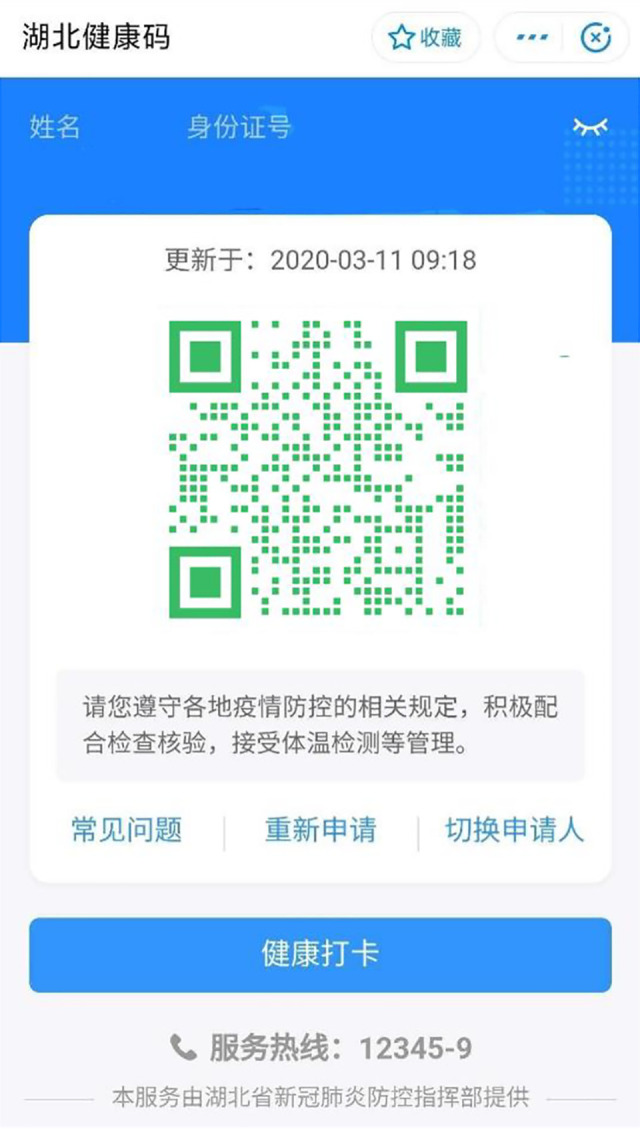
User interface of the Hubei Health Code (Green). *Note*: Screenshot was provided by an interviewee. His name and ID card number were removed by the author. The content of the original QR code was replaced with the color stayed the same.

Health Code operates similar to the passport. Once initiated, the Health Code in most places will generate a color code for individuals based on its algorithm’s evaluation of his/her risk status. There are three colors: green, yellow, or red. Green means “nonrisk,” while the other two mean “risky” at different levels (Figure 1). The use of Health Code in everyday situations follows an “inspect-pass/stop” procedure. At the entrance to spaces such as an office building, residential community, or supermarket, a checkpoint staffed with inspectors is set up. People passing through the entrance are required to show his/her Health Code risk status to the inspector. Those with a green code are allowed in, while those with yellow or red codes are stopped and reported to the disease control department. A “scan” procedure is required before the inspection in some cities/entrances, where specific a QR code registered for that location is set up. People passing the entrance are required to use the Health Code APP to scan the QR code for that entrance. Health Code uploads this check-in information to its server to document people’s movements and sends back a real-time risk status, following by the same “inspect-pass/stop” procedure stated above.

Complicated algorithmic systems depend on the imbrication of various devices and layers of infrastructures to function (Singh and Jackson 2017; Star 1990). Health Code is no exception. It operates as a mobile APP or on other APPs. It requires smartphones’ sensors and operating systems, which need cellular or Wi-Fi signals to upload the data and download the risk status from the algorithms in the cloud. Finally, the phones that Health Code runs on need to be charged. Although it may seem trivial to list them, these infrastructures and devices are fundamental parts of the Health Code’s sociotechnical assemblages. After all, when things work well, the functional infrastructure often remains hidden and taken for granted (Lampland and Star 2009). In 2019, out of a population of 1.4 billion, 897 million Chinese had phones that could access the Internet, and more than 80 percent had WeChat or Alipay (China Internet Network Information Center 2020). For modern Chinese, it is hard to imagine life without any of the infrastructures or devices listed above. As Cuiping, a bank manager, said, “my hands are always on my phone […] payment, work, life, contact, everything!” Later, I will show the potential problems generated by this taken-for-grantedness.

### Mobilizing People and Rearranging Space

Health Code mostly operates to record location history and assess risk status at checkpoints located at the boundary between spaces. For places with clear physical boundaries, limited entry, and mature security systems or inspectors, it is easier to enact its operation. For example, Chinese urban residential communities that were built after the 1990s are often gated with multiple entries. Many interviewees who lived in the gated communities told me that staff at the property management offices or community governments have blocked most of the entries during the pandemic. Only a few have been left open and secured by human inspectors to check passengers’ Health Code status. Similar boundary management happens in other spaces like supermarkets, buses, or office buildings, where the staff become the inspectors.

However, constructing a suitable environment for Health Code is not this simple for all spaces, as boundaries between many places are open or unclear. Even for the Chinese state, with operational apparatuses on almost every street, it is impossible to inspect all open boundaries. Haiyan, for example, observes that “the communities nearby my office were built in the 1980s; they’re just some independent residential buildings, and people can easily go everywhere from anywhere.” Space needs to be rearranged. For example, in the middle of February, Caixia, a community government staff member, was ordered by her supervisor to work as a Health Code inspector in her residential community. She had to use iron fences to block her old residential community’s open boundaries in order to approximate the effect of a gated one.

People do not naturally cooperate with Health Code; they need to be convinced and supervised to enroll and maintain its assemblage, which requires human labor. State institutions and their staff are the most important actors in this stage. They train nongovernmental security people from well-bounded spaces to become Health Code inspectors or they become inspectors themselves. In many places, community government staff go door-to-door in their community to introduce people to Health Code. Consent-building and stabilizing people’s enrollment is not easy. The rearrangement of spaces faces many challenges. Caixia complains that, particularly at the early stage of the pandemic, people were very uncooperative and “continually broke or jumped across the fence.” Extra staff were needed to patrol and maintain this rearrangement. She further explains:

The virus was not spread out in my city, so people didn’t feel the urge to do this extra work (using Health Code). People were complaining and even refusing to use it. […] there is nothing much we could do except talk them through. […] Most people do listen. We are all neighbors anyway. And we also had police come along. They never really arrest anyone, but the uniform helps.

During the pandemic, many municipal governments enforce administrative regulations that punish people who are uncooperative with disease control. The presence of police, even symbolically, is critical. However, as Caixia’s comment indicates, state power and law enforcement are not the only tools they used to enroll people and stabilize the assemblage; government staff also use their personal connections to persuade others (“we are all neighbors”).

This section shows how Health Code is assembled in society. Unlike in the popular imagination or even the Chinese government’s own advertising, automated contact tracing does not happen automatically. Surveillance technologies also do not work without human surveillance. Health Code is an example of how mundane but exhaustive work is often the prerequisite for an algorithm to unfold in society (Amelang and Bauer 2019; Lee et al. 2019), as it relies on countless instances of labor, the engagement of various actors, and a reorganization of society to assemble and stabilize the assemblage. Particularly, this process highlights how the enrollment of ordinary people into the algorithmic assemblage of a state’s legibility project is not a given; it must be achieved (Greenberg 2021; Rodríguez-Muñiz 2017).

## Destabilizing Health Code

### Breakdowns

Health Code’s status is updated frequently,^2^ requiring a stable Internet connection and GPS signal that are not available everywhere. Ningning regularly experienced problems using Health Code due to the location of her office building’s entrance: “I go to my office through the underground parking lot, where the signal is really bad. It always takes me forever to get Health Code; and I was almost late several times.” Even when all these infrastructures work well, one’s phone might not. A common problem is a phone’s battery running down. One time during her lunch break, Ningning left the building to pick up a delivery, and during this time, her phone died. “The security asked me to show him my Health Code. I said, ‘my phone just died!’ and he kept saying ‘you cannot get in if you do not have Health Code.’ He just saw me leave five minutes ago!” Only after twenty minutes of negotiation did the inspector finally let her inside. Furthermore, cell phone performance is also sometimes influenced by environmental conditions like the weather. Some interviewees experienced phone freezes during cold weather and could not open Health Code. These experiences illustrate the inconvenience one encounters if their phone is not functioning. This creates anxiety among some people when their phones are not on their person or fully charged.

### Dysfunctions and Misjudgments

Even when the infrastructure and devices that support Health Code function well, the Health Code algorithm itself can malfunction. For example, on February 20, Hangzhou’s (Zhejiang province) Health Code went down during the morning rush hour. One month later, both Hebei provincial and Kunming (Yunnan province) municipal Health Codes were out of service for hours. These crashes caused huge mobility issues and delays. Wenhong experienced three crashes. To pass the inspectors during the crash, she had to fill out her ID card number, name, phone number, and address manually on the register sheet that were provided by her residential community or subway stations. In other places, inspectors simply refused to let people into the space they guarded during the Health Code crash.

These systemwide failures are consequential but also rare. More frequently, Health Code only became mildly dysfunctional, for example, processing slowly or preventing users from logging in or scanning the QR codes. Dawei complained that his provincial Health Code lacks optimization: “it usually takes me half a minute to process the Health Code scanning process, and I am using an iPhone! Can you imagine how slow it would be for people using an Android?”

Health Code also makes errors assessing people’s risk status. This was particularly common during the early period of Health Code implementation. In mid-March, Qiangzi, a college student in Tianjin, found his Health Code color turn red for no reason. As usual, he scanned the QR code at his residential community and showed his screen to the inspector, “I can see his face suddenly change color, and that’s when I realized my Health Code’s color is not green.” He jumped the wall of his community and went back home, as the inspector refused to let him in. Then, he immediately called the Health Code contact number.

I was pretty sure I didn’t go anywhere risky […] The representative said she can only help me to file the appeal and wait for other staff to check and did not tell me what was wrong. I know I cannot go out. If I am not green, then I cannot go anywhere anyway. I was afraid if my neighbors saw police or doctor came to my door and spread gossip about what I did. I freak out even when someone knock on my door. […] I waited and waited. At 2 am, it suddenly became green again.

Some believe that having an algorithm with false positives is better than false negatives during the pandemic; “better safe than sorry” is a common reaction among those people. However, for those who experienced misjudgment like Qiangzi, the whole process was confusing and terrifying. Although eventually resolved, no explanation was given regarding this incident, creating uncertainty. Experiencing misjudgment incidents, or just knowing about any such incident, changes people’s trust of Health Code and makes them more careful in interpreting their social surroundings. Besides, no one is certain that the algorithm only produces false positives. After her father was misjudged by Health Code, Linda started to be suspicious about the risk status of people in public spaces, all of whom should have a green Health Code and low risk. “Who knows if they are really low risk? […] I really lost my confidence. The system seems to be quite unreliable now.”

### Exclusion

Even if Health Code works perfectly, it still excludes certain groups. Although all of my interviewees have no trouble using Health Code, most of them know someone who is excluded by it. The elderly are a commonly excluded group, not only because of the lower percentage of smartphone ownership but also the lower capacity to navigate through Health Code’s digital interface. Nana’s grandfather, for example, stayed home for months: “It is easy for us young people, as we scan stuff all the time, yet it was confusing for him. […] He’s ok because he can stay at home, and my parents brought groceries for him. But some people have to get out, and now they are excluded.”

Poor people who cannot afford a phone or data plan are also excluded by Health Code; therefore, they are excluded from most public spaces and public services. Ironically, they might rely more on public goods, such as buses or subways, while affluent people can drive. For example, Hanhan, a young man who lost his job in the Netherlands and came back to Guangzhou (Guangdong province) had a memorable experience:

I spent most of my earnings to repay debts and did not have much when I got back to Guangzhou, and I didn’t know I needed to pay for the quarantine at my own expense. So, when my quarantine ended, I didn’t have much cash left, and my phone broke. I cannot go anywhere but my quarantine hotel because I didn’t have a phone for Health Code. I cannot stay because I didn’t have money left. […] I ended up walking from the hotel to the city center with a small map I got from the hotel for more than 20 kilometers. […] it is easy for people without any special condition, but this is so unfriendly for anyone with special needs.

Hanhan’s experience highlights the hidden assumptions of normalcy in Health Code’s implementation—possession and maintenance of a working phone—during this abnormal time. These exclusions contradict impressions of a ubiquitous and convenient technological disease control solution, which also undermine Health Code’s legitimacy among the included. People lament for the excluded and, therefore, doubt whether Health Code is appropriate. Some argue that the system is discriminatory and unfair. Others argue that the exclusion of specific groups of people may impact the usefulness of the algorithms. For example, Nana asks, “Eventually, is Health Code collecting data from everyone or just a specific group that has good economic status and is educated?”

This section shows how Health Code’s algorithmic assemblage is destabilized by errors, challenges, and breakdowns, as well as the exclusion of citizens from its operation. China’s strong state capacity and robust infrastructural base afford a quick assembly of Health Code for most people and in most places. However, neither state capacity nor infrastructure coverage can be comprehensive (Greenberg 2021; Houston, Gabrys, and Pritchard 2019; Vertesi 2014). As studies on how one’s infrastructure could be another’s barrier have shown, an algorithm’s inclusion and exclusion are always relational (Lampland and Star 2009; Star 1990).

## Reassembling Health Code

### Elastic Surveillance

Instead of worrying that intensive Health Code surveillance would advance state control, most interviewees worry that Health Code’s surveillance is *not* implemented strictly enough. Commonly, people found that inspectors do not carefully check their Health Code. Junzi, a documentary director who traveled to more than ten cities during the pandemic, states that “Health Code is useless for 95 percent of the places I visited. People do not check if you really scanned the entry code or not.”

Scale is always a problem for countries with a huge and dense population like China. When Health Code was first proposed, most people were required to work or attend school at home. As society reopened, the population started to circulate again, which increasingly overburdened the inspectors. As Qiangzi reflects, “We have 1.4 billion people living in China. For metropolitan cities like Beijing, Shanghai, and Guangzhou, even if Health Code works super-fast, how many people are in a subway station during rush hour? Even if everyone takes just two seconds—which is not really what is happening now—how many hours are wasted on just opening it?”

Besides undercapacity, other social incentives also undermine Health Code’s ideal operation. Disease control is not the only priority of social actors. Runze, for example, observes that many supermarkets or small businesses do not check Health Code at all. “They want to do business, of course. They want, as much as possible, people to come in and not slow down the crowd.” Relational factors also play a role in reshaping Health Code’s implementation. Qiangzi finds that his neighborhood inspector is very friendly and often skips checking residents he knows personally. This social exception extends to groups also. As Lulu, an employee at a Shanghai media company notices, “There’s a wild card for Health Code checking: Shanghai dialect. If you speak Shanghai dialect to the inspector (of her residential community), you can just go without being checked.”

Finally, this elastic surveillance is sometimes purposely enforced to help people who are excluded by Health Code or when Health Code goes wrong. Alternative methods include certifying one’s health status and travel documentation using pen and paper. For example, the community government where Caixia works could issue health certificate cards to elderly people for going out without using Health Code. However, this practice requires that elderly people bring their ID cards with them at all times and write down their personal information whenever they enter public spaces. These measures are also not recognized universally. Lisha’s grandmother, for example, used the community government’s certificate to get out of her community, yet she was not allowed to get into the subway because they only recognized Health Code at that location.

### Extra Scrutiny

Another common situation that deviates from Health Code’s design occurs when local authorities favor alternative risk evaluations and control practices over Health Code’s. People from Wuhan city or Hubei province, where the pandemic started, are commonly under this kind of stricter scrutiny. Runze is a journalist who lived in a city near Wuhan during the lockdown. When his Health Code turned green after one month’s quarantine, he received a message from local government staff claiming, “Yes, the Health Code is green, but that was produced by ‘big data’. It will still depend on local policy and interpretation to determine if one can go out.” The local government required him to stay at home for another two weeks. Runze went back to Guangzhou at the end of March, and his Health Code remained green the whole time. However, the inspector at his residential community’s entrance maintained extra distance from him after they found out he was from Hubei. Police were called, and the local government requested that he fill out many additional questionnaires and daily reports regardless of his green Health Code. For two months, as he tried to report government events or conduct interviews, people simply refused him access once they learned that he was from Hubei, even though he always told them his Health Code status first.

Generally, additional evaluations are more likely to be applied to community outsiders. Personal ID cards or car plates are used by authorities to identify people who come from other cities. Yuejin, a retired high school teacher, drove for two days from Harbin (Heilongjiang province) to Qingdao (Shandong province) to see his children in March, when an outbreak happened in Harbin.^3^ Both his Harbin and Qingdao Health Code statuses were green, which helped him pass his residential community’s checkpoint in Qingdao. However, some residents saw his Harbin car plate and reported him to the community government. The government sent five people the next day, asking him to quarantine at home for two weeks despite his Health Code status. He argued that his risk status was scientifically calculated by artificial intelligence and big data, to little effect: “They are so uneducated!” Eventually, he had to call Qingdao Center for Disease Control to validate his Health Code status.

This additional scrutiny is associated with a specific political environment. After the Wuhan lockdown, controlling the spread of the virus became extremely politicized. As Hanhan comments, “no one wants to have COVID in their jurisdiction.” Upper levels of government enforce increasingly strict requirements on lower governments to control the outbreak. In many places, senior government officials of areas with new outbreaks are held personally accountable for outcomes and are liable to be punished by the party and national government. This results in extreme caution among local authorities, inclining them to determine that the risk status produced by Health Code alone is insufficient.

Although Health Code is constantly reassembled in the situations described in the previous sections, these reassemblies are mostly responsive and intended to reconfigure Health Code back to its ideal design and assemblage. In other words, reassembly happens through problem-solving. The reassemblies in this section, however, are more the result of intentional deviations; therefore, they are more irreconcilable and salient in their ontological multiplicity (Mol 2003). As studies of other algorithms have shown, reassembly is particularly sensitive to the existing power hierarchy and organizational logics (Christin 2020; Brayne 2020); thus, it impacts both how the data fed into the algorithm are collected and how the output from the algorithm is interpreted.

## Bypassing Health Code

In dealing with all of the breaks, errors, and deviations in Health Code’s assemblages, people’s perceptions of and practices using Health Code changed. Many of them began to bypass the system for different reasons. The most common way to bypass Health Code is to use a screenshot. For many, it is simply a convenient practice to skip annoying APP loading times due to a weak signal or dysfunctional platform. Lulu, for example, complains that “it is really annoying to initiate Health Code in winter when your phone is particularly slow loading the camera, and with all the people in front of you trying to find an angle to scan the QR code. […] I don’t want to waste my time. It’s useless anyway.” As a result, Lulu used a screenshot of her own green code for several days to skip the line. Many municipal authorities noticed this bypassing and upgraded their Health Code’s color code interface from static to dynamic with a live countdown clock in the corner. This does not stop people from gaming the system. At least one fake Health Code APP that generates an offline and customizable Health Code dynamic interface was created and used.^4^ Moreover, even when most APPs were upgraded with the dynamic risk status interface, screenshots remained a common practice due to inspectors’ lax enforcement.

People also upload fake information to Health Code. Some people do this to avoid the tiring and repetitive processes of filling in the same information on different platforms. Lingling traveled to four cities during April and had to initiate four Health Code systems. As data from different cities’ Health Codes are not connected, she had to fill in the same questions multiple times: “I do not know why they cannot share the data, but I lost my patience eventually and filled some random stuff in.” Dawei recalled his tourism trip to Chongqing, a city that is two hours away from his resident city by flight: “When I arrived, the staff started to ask you to initiate two Health Codes, one is the State Council’s, and another is the Chongqing one. It was so annoying. My phone was slow, and people were lining up, so I just filled something out and made up the rest to save time.” Others upload fake information to avoid being evaluated as high-risk and forced to quarantine. Some of these come from real concerns that they may be infected, while the rest are concerned about the algorithm’s potential misjudgment. For instance, Lisha, a young law student, struggled when she initiated her Health Code and eventually filled in a fake answer: “I have chronic sore throat and cough for months. Should I fill that I have the symptoms? Of course not! If I said that, who knows what will happen and how much trouble it might cause?” She doubted that the algorithm could differentiate her chronic problem from COVID-19 symptoms with the decontextualized symptom questionnaire.

Furthermore, some people actively seek out corners that are not covered by staff checking Health Code. Kailing and her mother both exit their residence via an underground parking lot where no inspectors are located in order to save time. When her friend, Junzi, came to visit, they tried to find places where staff were not enforcing Health Code strictly, as Junzi worried that a record of visiting Guangzhou might elicit forced quarantine upon his return home. He says, “For many restaurants, they only check one person’s Health Code if you are a group. So, we will only go to places like that with me scanning Health Code. If the waiter intends to check everyone’s, we will just leave and find another restaurant.” These practices of bypassing, avoiding, and gaming the algorithm further complicate the reassembly process, sometimes even disassembling parts of Health Code’s assemblage. Aimi encountered a bus driver who did not check her Health Code in April. She asked why, and the driver responded, “What’s the point of doing this? People made the information up.”

This section illustrates how ordinary people flexibly navigate the state’s algorithmic surveillance and evaluation regime, echoing other studies on human agency under various algorithmic regimes and state legibility projects (Chen and Sun 2020; Greenberg 2021). These gaming practices are not necessarily resistant in consciousness but are often situationally spontaneous. Nevertheless, they challenge deterministic imaginations of a stable, centralized, and top-down control granted to an authoritarian state by the algorithm.

## Discussion

Analyzing the assembling of Health Code during the COVID-19 pandemic provides a unique opportunity to examine how states construct and operate legibility projects with algorithms. The case of China meets three conditions of power concentration: an authoritarian state, using algorithms, under a state of emergency. Adding these together, one may expect a story of crystal clear legibility. This study does not deny the power concentration and increased legibility accorded by the trio nor does it attempt to claim there are no risks or costs to such operation. Health Code undoubtedly shows how a state can quickly expand its tentacles for seeing and intervening in society as well as how people’s everyday life can be reorganized based on their positionality within algorithmic evaluation. However, this legibility has considerable limits even under all these favorable conditions. I offer a more nuanced and realistic way of understanding how the state sees from how the algorithm is enacted.

Health Code’s sociotechnical assemblage is entangled with heterogeneous social and technical elements beyond its technical designs. It connects with and enrolls diverse human and nonhuman actors, such as government bureaucrats, community volunteers, programmers, iron fences, smartphones, and so on. Political pressures, commercial incentives, interpersonal relationships, and regional affinities are also always involved. As a result, Health Code ontological boundaries are constantly being modified as it is reconfigured across time and space, generating multiple Health Codes even within the same Health Code system. One person might experience lax surveillance in her community because the inspector is someone she knows, only to then be blocked at a hotel, despite a green Health Code risk status, because her ID card is registered from Wuhan. These multiplicities and messiness are not the result of the algorithm’s technical design nor a simple reflection of weak enforcement capacity; therefore, they cannot be solved by a perfect design or protocol. Ironically, the more insistent and well-rounded the design of an algorithm is, the more complicated, flexible, and fluid the social processes must be to achieve the algorithm’s stabilization.^5^ Such accommodation requires the engagement of more diverse social actors. In between these multifaceted interactions, people are not fully controlled by the algorithm as uniformly compliant subjects; rather, they are continually looking for loopholes in the sociotechnical assemblages so as to bypass its regime. These dynamic, processual, and contextual assemblies, disassemblies, and reassemblies of an algorithm can only be demonstrated when we think and research algorithms beyond the blackbox metaphor, focusing on how an algorithm enacts and performs in broader sociomaterial settings.

The application of Health Code in the pandemic highlights a new way of seeing like a state, which is about not only improving the scale and details a state can expect to see but also changing how it sees and intervenes. Unlike the modern state in Scott’s conceptualization, which abstracts society with representations, sees people as populations, makes plans based on the abstractions, and then implements the plans, the emerging “dataist state” collects real-time data and makes evaluation and interventions on individual bases. Moreover, it constantly reconstructs its vision based on feedback (Fourcade and Gordon 2020; Johns 2019). Health Code’s operation is largely the instantiation of this new paradigm. However, it is noteworthy that the transformation of statecraft does not happen in a comprehensive and once-and-for-all manner, as the state itself is often fragmented in a way that requires specific analysis (Jasanoff 2004; Morgan and Orloff 2017). The dataist state logic also coexists with the modern state’s traditional ways of seeing, evaluating, and controlling, which was shown in those cases when the local government refuted Health Code’s algorithmic evaluation and insisted on evaluating risk based on place of origin. Constructing and maintaining algorithmic legibility is also by no means automated and human-free, as many people claim. On the contrary, like assembling other algorithmic systems or state legibility projects (Geiger 2017; Greenberg 2021; Rodríguez-Muñiz 2017), assembling the state’s algorithmic vision requires intensive and invisible human interventions. Space is reorganized, people are rearranged, and various devices and infrastructures are constructed and connected.

These efforts do not stop when the sociotechnical assemblage is established. They continue due to ongoing complications as the algorithm further connects with more diverse social actors and infrastructures. After all, the wider an algorithm covers social life, and the more actors an algorithm connects, the more opportunities for things to go wrong, resulting in a chain reaction that destabilizes or distorts the assemblage. To reduce such risks, more supervision, inspections, and regulations are established because formalizing systems always require arduous and flexible work to be maintained (Lampland 2010). Yet errors, exclusions, and challenges emerge continuously. Of course, patchwork follows, algorithms evolve, and cooperation improves, resulting in higher stabilization of the algorithmic assemblage.^6^ However, the risk is not only about the likelihood of occurrence but also of consequences. With the increased embeddedness of algorithms in social life and the state’s higher level of dependence on its algorithmic vision, such destabilization and distortion could immediately have a larger impact on a great number of people (Singh and Jackson 2017), sometimes secretly (Eubanks 2018). In other words, the more important an algorithm is, the more people rely on it, the more risks it generates. The dataist state shares characteristics similar to the modern state’s way of seeing citizens: top-down, expert-centered, and hierarchical (Fourcade and Gordon 2020). While the dataist state can achieve a level of vision that the modern state cannot, it also faces the increasingly complicated and deeper risks of being interrupted and blinded.

The state’s algorithmic vision poses new questions about how the state recognizes citizens, as well as how citizenship is claimed in the digital age, where a citizen’s position in the state’s algorithmic vision is increasingly critical (Fourcade 2021). In this case, elaborate surveillance citizenship has also emerged: People must be surveilled and datafied by specific platforms to access certain public spaces or services. Even when we acknowledge the public’s perception of the surveillance as proof of caring (Liu 2021; Liu and Graham 2021) and recognize algorithmic assemblages are multiple, elastic, and porous, this caring and flexibility do not apply to everyone equally. As we have observed, some people game Health Code to save five minutes waiting in a line, while others are stuck at home for weeks due to confusion about the technology. Algorithms, even in their deviations, always have the potential to damage some people, often marginalized groups, more than others. Such inequality-generating mechanisms have been observed in other everyday infrastructures, policing, and social welfare across the world (Brayne 2020; Eubanks 2018; Pasquale 2015).

Of course, unequal citizen rights based on an unequal inclusion in state legibility projects are not new. Nevertheless, due to the accelerating expansion of algorithmic systems in social life and the risks stated above, the effects of the new form of inequality may be more significant than before. The digital inequality problem itself has also become more complex than the classical digital division problem of access. As this study and other scholars have shown, digital inequality is now a result of differences not only in access but also in the capacity to navigate across different and ever-changing algorithmic systems (Fourcade 2021; Singh and Jackson 2017). However, these increasing effects and the complexity of digital inequality are often overlooked as digital infrastructural coverage continues to expand and ownership of smart devices continues to increase. One way to address this problem is to make the ignored legible in the state’s algorithmic eyes, similar to the inclusion movements for financial credit records and census projects (Rodríguez-Muñiz 2017; Rona-Tas 2020). However, we also need to think beyond the state’s vision and consider citizens beyond their status subjects. As Fourcade and Gordon (2020, 98) urge, we should think about a vision of “seeing like a citizen,” a collective form of governance, “turning that scrutiny back upon the state and politicians themselves.”

The state of emergency caused by COVID-19 will fade, and the algorithmic assemblage assembled in response to the pandemic may be disassembled. However, the infrastructure built, connections made, and norms established for the state’s algorithmic eyes may persist. Social scientists must carefully identify and challenge the normalization and institutionalization of these pandemic infrastructures, connections, and norms that have the potential to reshape postpandemic institutions, social orders, and everyday life. We must reject two extreme versions: dystopian determinism on the one hand and blind faith in technologies on the other. We must confront the claim that algorithms and other technologies are universally beneficial by critically examining the exclusions, inconsistencies, and contradictions they foist upon social life without falling into an oversimplified fatalist narrative. Detailed and even “boring”—as Lampland and Star joked (2009, 17)—empirical works that examine how algorithms and states work in action are urgently needed. These tasks go beyond the context of China and are valid far beyond the COVID-19 pandemic.

## Appendix

**Table A1.** List of Interviewees.

Name	Gender	Location	Age	Occupation
Dawei	Male	Anhui	26	Graduate student (in design)
Junzi	Male	Beijing	42	Self-employed (filmmaker)
Hong	Male	Fujian	26	Graduate student
Runze	Male	Guangdong	24	Private company employee (journalist)
Kailing	Female	Guangdong	31	Foreign company employee (researcher)
Xiangzi	Male	Guangdong	26	State-owned enterprise employee
Anan	Female	Guangdong	26	Non-governmental organization employee
Hanhan	Male	Guangdong	27	Part time worker
Gangzi	Male	Guangdong	35	Private company employee (psychologist)
Meilin	Female	Guangdong	23	College student (in medicine)
Jianguo	Male	Guangdong	52	State-owned enterprise employee
Feng	Male	Guangdong	26	Private company employee
Huadong	Male	Hubei	21	College student
Niuniu	Female	Heilongjiang	22	College student
Haiyan	Female	Heilongjiang	36	Public institution employee
Cuiping	Female	Heilongjiang	49	Public institution employee (bank manager)
Wenhong	Female	Heilongjiang	25	Private company employee
Xiaoli	Female	Henan	18	High school graduate
Pingping	Female	Hunan	18	High school graduate
Feifei	Female	Jiangsu	26	Graduate student (in law)
Lisha	Female	Jiangsu	25	College student (in law)
Wutong	Male	Jiangsu	23	College student
Longzi	Male	Jiangsu	25	Graduate student (abroad)
Yuejin	Male	Shandong	55	Public institution employee (retired teacher)
Caixia	Female	Shandong	50	Community government employee
Lingling	Female	Shanghai	32	Foreign company employee
Wenzi	Female	Shanghai	34	Private company employee
Paoge	Male	Shanghai	27	Private company employee
Yanzi	Female	Shanghai	22	College student
Lulu	Female	Shanghai	27	Private company employee (media related)
Xiaowang	Female	Shannxi	20	College student
Donglin	Female	Shannxi	24	Self employed
Linda	Female	Sichuan	27	Private company employee
Qiangzi	Male	Tianjin	21	College student
Ningning	Female	Zhejiang	24	Private company employee
Aimi	Female	Zhejiang	37	Private company employee (technology related)
Nana	Female	Zhejiang	23	College student
Pink	Female	Zhejiang	22	Private company employee

## Note

1. For example, location mattered because Health Code was designed and distributed by different municipal and provincial governments. Although socioeconomic status (SES), such as education and income, also played a critical role in experiencing Health Code, I was concerned that they will be sensitive questions to ask in order to filter people for participation. My reliance on online recruitment also restrained my ability to reach out to low SES populations. A more detailed methodological reflection about their exclusion from digital studies was published in another paper (Liu and Graham 2021)

2. How often the Health Code status updates is not clear. None of my interviewees were sure about it, while official statements vary from every time an individual opens the APP to weekly.

3. The distance between Harbin and Qingdao is approximately 1,700 kilometers (≈1,060 miles).

4. The developer of the fake APP was quickly arrested, and the APP was deleted (Ma 2021).

5. Such irony is also seen in Lampland’s (2010) argument about formalizing practices.

6. In fact, it is understandable that Health Code algorithms malfunction sometimes, especially considering the limited time and resources for their development and implementation. The first Health Code was developed in two days for Hangzhou’s 10 million population (Zhang and Tang 2020).
